# Lymphopenia without neutropenia in Clozapine treatment. A review and a case report

**DOI:** 10.1192/j.eurpsy.2025.2096

**Published:** 2025-08-26

**Authors:** C. Munaiz Cossío, I. M. Peso Navarro, N. M. Casado Espada, M. Ligero Argudo, R. K. Gonzalez Bolaños, C. Payo Rodriguez, C. Garcia Cerdan, P. Andres Olivera, C. Puerta Vazquez

**Affiliations:** 1 Psychiatry, Complejo Asistencial Sanitario de Salamanca; 2 Psychiatry, Hospital de Salamanca; 3 Hematology, Complejo Asistencial Sanitario de Salamanca, Salamanca, Spain

## Abstract

**Introduction:**

In 1975 Clozapine was retired after 16 cases of severe neutropenia, with a mortality of 50%. It wasn´t until 5 years later when its effectiveness in treatment resistant schizorenia, with a mandatory hematological follow up.

In studies available we find that clozapine treatment is related to neutropenia and not leukopenia. In the case we present below neutrophils are within range, but it´s lymphocytes that are affected.

**Objectives:**

We hope that our expirience, and review can help other professionals in the future who find themselves in this situation.

**Methods:**

We used the Pubmed and Uptodate databases.

We present the following clinical case.

Male, 36 years old, with a diagnosis of Schizophrenia. Several admissions to the Acute Unit over the years, requiring treatment with ECT. Maintenance treatment with Olanzapine, with which he maintained some delirious ideation and tendency to isolation. He was admitted again in 2023 due to a destabilization of his pathology, presenting delusions of harm, persecution, self-referentiality, auditory hallucinations, imperative phonemes, etc. with important affective and behavioral repercussions. Several pharmacological treatments were tried (Olanzapine, risperidone, aripiprazole), finally the patient showed some improvement with Lurasidone although his functionality was still impaired.

It was decided to start treatment with Clozapine, to minimize the psychotic symptoms, after a hemogram study, which was normal.

**Results:**

During the weekly follow-up of the treatment, a decrease in lymphocytes was observed, with normal neutrophils. The treatment was proving to be ineffective, so it was decided to continue in this line.

Seven months after starting the treatment, the patient suffered a catarrhal process, and once resolved, we observed in addition of the lymphopenia, anaemia and grade 2 neutropenia in the hemogram. Succeeding a consult with hematology specialist we decided to stop the treatment.

The week following the suspension of the treatment, the hemogram normalizes, but the psychotic symptomatology worsens (inability to relate to others, thought blocks, etc.). Taking into account that the blood alterations occured after a cold, and the mental deterioration that the patient presented, it is agreed with the family and the patient to restart the treatment. Wich resulted in improvement of the psychotic symptoms but a new leukopenia due to a slight lymphopenia is observed again.

**Conclusions:**

The average time described for the resolution of severe neutropenia is 12 days. In our case, the hemogram started to improve by the fifth day following the suspensión of the treatment. As it is an infrequent side effect, we do not have studies on the effects of lymphopenia secondary to Clozapine.

We decided to mantein the Clozapine treatment due to the great improvement of the patients quality of life.

Currently he is taking Clozapine 75mg a day and remains stable.

**Disclosure of Interest:**

None Declared

